# Laser Joining of Continuous Carbon Fiber-Reinforced PEEK and Titanium Alloy with High Strength

**DOI:** 10.3390/polym14214676

**Published:** 2022-11-02

**Authors:** Haipeng Wang, Zhongjing Ren, Yingchun Guan

**Affiliations:** 1 Key Laboratory of High-Efficiency and Clean Mechanical Manufacture of MOE, School of Mechanical Engineering, Shandong University, Jinan 250061, China; 2 School of Mechanical Engineering and Automation, Beihang University, Beijing 100083, China

**Keywords:** laser joining, fracture strength, heterojunction, mechanical interlocking structure, CCF30/PEEK

## Abstract

The generation of high-performance heterojunctions between high-strength resin matrix composites and metals is of great significance for lightweight applications in fields such as aerospace and automobile engineering. Herein, we explored the feasibility of employing a laser joining process to achieve high-strength heterojunctions between continuous carbon fiber-reinforced PEEK (CCF30/PEEK) composites and titanium alloy (Ti6Al4V). A joint strength of over 50 MPa was achieved through constructing mechanical interlocking structures between CCF30/PEEK and Ti6Al4V. Tensile tests revealed that the fracture of joints was mainly ascribed to the detachment of carbon fibers from the resin matrix and the breakage of carbon fibers. The structures with different orientations and dimensions were confirmed to significantly influence the formation of interlocking structures near the joining interface and the resultant fracture strength of joints. It is believed that the results presented in this study provide a strong foundation for the production of high-performance heterojunctions.

## 1. Introduction

Lightweight materials are the eternal goal of the aerospace field, where even the reduction of each gram is rigorously considered [1,2,3,4,5]. As the applications of resin matrix composites, mainly carbon or glass fiber-reinforced polymers (CFRP or GFRP), have increased in aerospace component manufacturing, the preparation of high-strength heterojunctions between metals and thermoplastics has become a major concern [6,7,8]. After decades of development, laser joining technology emerged from various joining technologies due to its advantages, such as non-contact methodology, high flexibility, high efficiency, and environmental friendliness [9,10]. Tan et al. [11] investigated the effect of laser power on the interface morphology and the performance of laser joining-prepared AZ31B-CFRP joints. The formation of diffusion layer and chemical bonding at the interface led to good bonding performance between the metal and CFRP. Elahi et al. analyzed the failure mechanisms of laser joining-produced Al-PA (polyamide) joints, and revealed that the adhesion at joining interface could be improved by laser ablating or polishing on the Al surface due to the formation of an artificial oxide layer [12].

The construction of mechanical interlocks between the metal and polymer has been a commonly used strategy to improve joint strength [13,14,15,16]. Jiao et al. [17] developed a hybrid surface pre-treatment method by adding a PA layer to the CFRP-Al alloy interface and machining microtextures on the Al alloy surface to enhance the joint strength. Owing to the increase in contact area and the generation of mechanical interlocks between CFRTP and the Al alloy, the maximum joint strength reached 37.5 MPa. Shan et al. clarified the formation mechanisms of porosity defects near the interface, and suppressed the shrinkage porosity by fabricating protrusions on steel to change the heat conduction path during the laser joining process and form mechanical interlocks at the joining interface [18]. The fracture strength of the resultant joints was increased from 9.3 MPa to 30.8 MPa. In our previous studies, an optimized laser joining configuration was designed to eliminate the porosity defects and strengthen the mechanical interlocks near the joining interface. The resultant joint strength between Ti6Al4V and GF30/PA66 improved from 13.8 MPa to 41.5 MPa [19,20]. The influence of structure density on the joint strength between Ti6Al4V and CF30/PEEK was further analyzed, and the obtained joints presented high fracture strength and excellent anti-aging properties, with the largest fracture strength reaching over 60 MPa [21].

Numerous studies have demonstrated the feasibility of laser joining technology in producing strong heterojunctions and revealed the bonding mechanisms near the interface of dissimilar materials [22,23,24]. However, the current studies mainly focused on the joining of short fiber-reinforced thermoplastics and metals; few studies have attempted to explore the connection between continuous carbon fiber-reinforced thermoplastics and metals through a laser joining process. Polyetheretherketone (PEEK) has excellent mechanical properties and has been used as a resin matrix for high-performance fiber-reinforced composites [25]. Composites containing continuous fibers display much higher performance than those containing short fibers, showing great potential applications in aerospace industry [26]. In this study, we explored the feasibility of laser joining technology in connecting titanium alloy and continuous carbon fiber-reinforced PEEK (CCF30/PEEK) composites. Before the laser joining process, various grating and grid patterns with different dimensions were pre-textured on the Ti6Al4V surface to obtain effective mechanical interlocking structures between CCF30/PEEK and Ti6Al4V. The maximum joint strength of 58.0 MPa was successfully achieved during tensile tests. The presented strategy provides good prospects for the production of high-performance heterojunctions between high-strength composites and metals.

## 2. Materials and Methods

### 2.1. Materials

**Figure 1:**
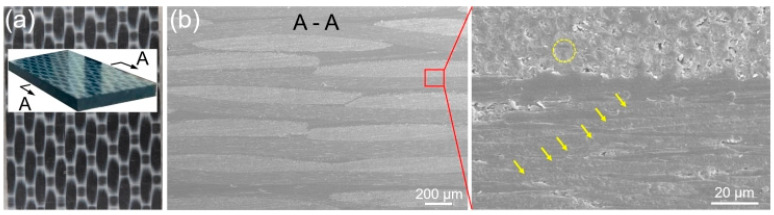
Cross-sectional microstructures of CCF30/PEEK sheet. (**a**) Macro image of the utilized CCF30/PEEK plates in experiments. (**b**) SEM images of the cross-sectional morphologies of CCF30/PEEK plates.

The employed substrate materials were the titanium alloy Ti6Al4V and continuous carbon fiber-reinforced thermoplastic composites, where the composites consisted of polyetheretherketone resin and 30wt.% continuous carbon fibers (CCF30/PEEK). Both the substrate specimens have a dimension of 20 mm × 100 mm × 2 mm. The melting point and the decomposition temperature of CCF30/PEEK are approximately 343 °C and 460 °C, respectively [26,27]. The CCF30/PEEK laminate, supplied by Jiangsu Junhua High Performance Specialty Engineering Plastics Products Co., Ltd., was prepared by hot-press molding of the impregnated tapes, where the tapes were laid in blending mode as displayed in Figure 1 and the thickness of each tape was approximately 100 μm. It is shown in Figure 1 that the layered impregnated tapes and continuous carbon fibers are clearly visible, and the impregnated tapes are tightly arranged. The carbon fibers are evenly dispersed and wrapped by polymer matrix, showing excellent interfacial adhesion between carbon fibers and polymers. The diameter of the resin-wrapped carbon fibers is around 5 μm, as indicated in Figure 1b. The tensile strength of CCF30/PEEK is about 560 MPa. Laser cleaning process was adopted to remove the oxide layer on Ti6Al4V surface before laser texturing [28]. The nominal chemical composition of the utilized Ti6Al4V is shown in Table 1.

### 2.2. Laser Texturing Process

**Figure 2:**
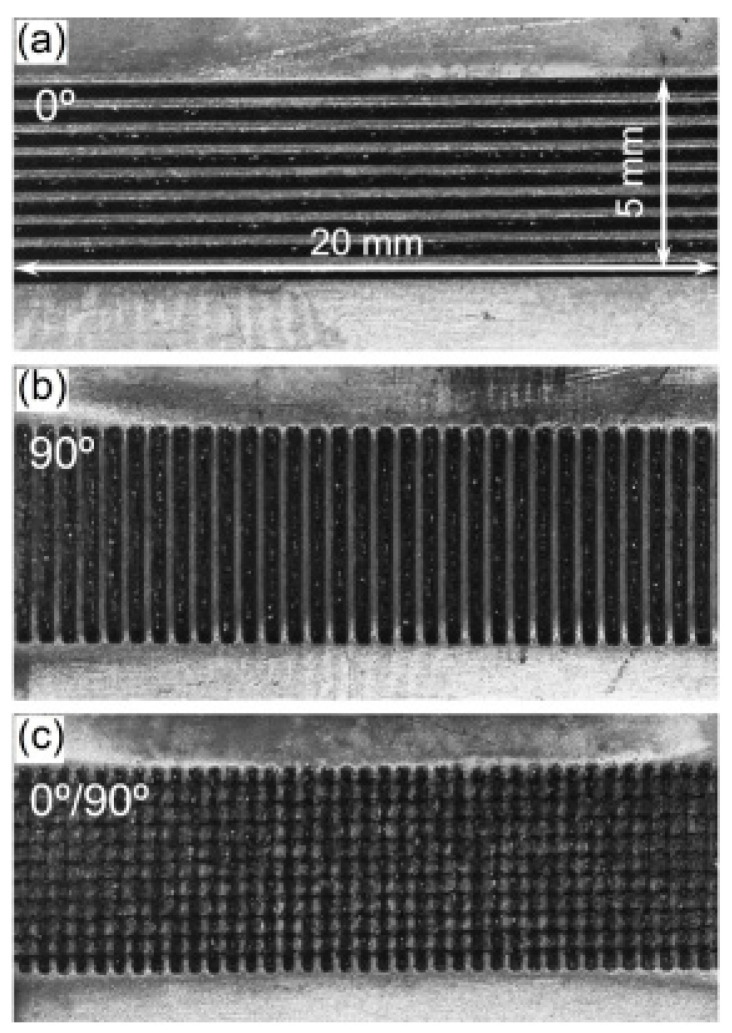
The different laser machined patterns of 0° grating (**a**), 90° grating (**b**), and 0°/90° grid (**c**) structures on Ti6Al4V surface.

**Figure 3:**
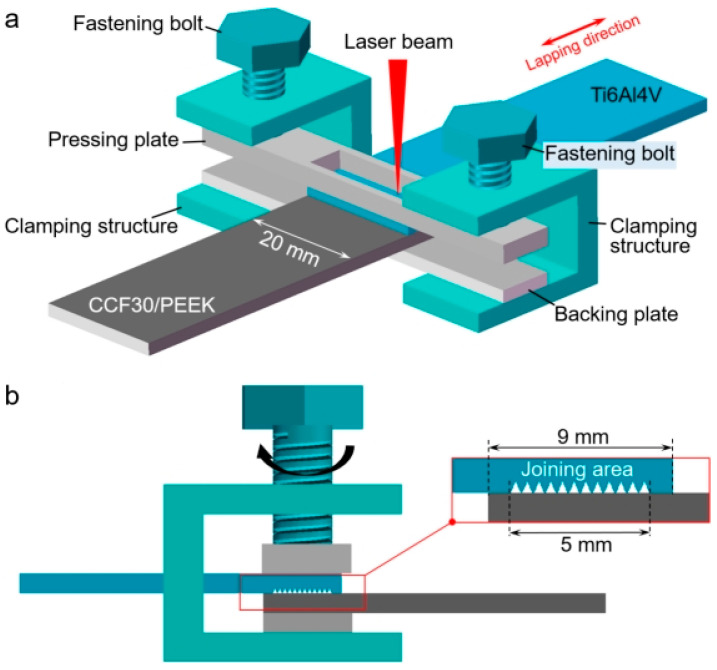
Schematics for laser joining of Ti6Al4V and CCF30/PEEK. (**a**) Diagram showing the laser joining process. (**b**) Diagram showing the lapping and joining area.

**Figure 4:**
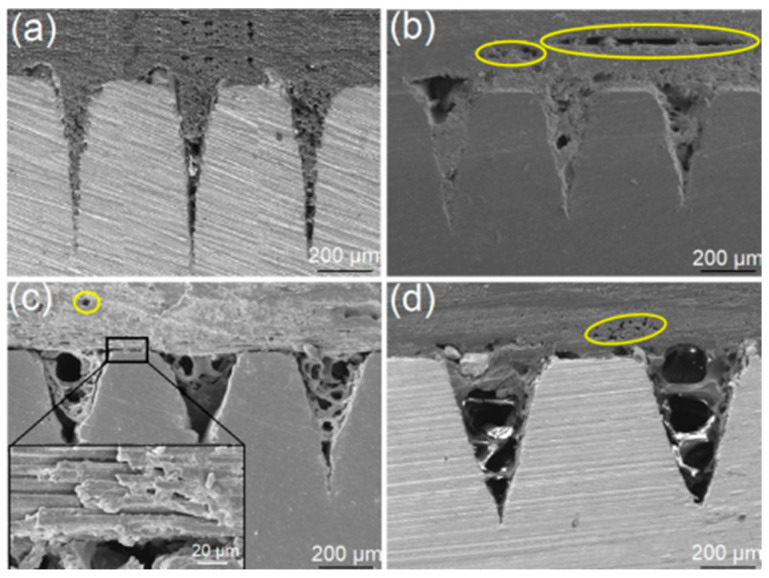
Cross-sectional morphologies of laser joining-produced CCF30/PEEK-Ti6Al4V joints in case of the 0° grating textures with microgrooves width of 225 ± 15 μm (**a**), 265 ± 10 μm (**b**), 335 ± 10 μm (**c**), and 425 ± 15 μm (**d**), respectively.

Ti6Al4V surfaces were textured using a nanosecond laser system (Time Bandwidth; Duetto) at a repetition rate of 55 kHz with a central wavelength of 1030 nm. The laser beam has a Gaussian profile with a TEM00 (M^2^ < 1.3) spatial mode, and is directed to the sample surface using a galvanometric scanner with a telecentric f-theta lens. To analyze the influence of laser parameters on structural dimensions, numerous pre-experiments were carried out by varying laser power, frequency, and travel speed. The optimized laser parameters for machining microgrooves are shown in Table 2. The area of laser textured pattern is 5 mm × 20 mm, as indicated in Figure 2. Three types of patterns, namely 0° grating, 90° grating, and 0°/90° grid, were fabricated on Ti6Al4V surface (Figure 2), where the orientation of the microgrooves in 0° and 90° gratings was perpendicular and parallel to the lapping direction of joint (Figure 3), respectively. Four groups of the structures with different dimension features were fabricated in each pattern as summarized in Table 3, where the structure density of these patterns ranged from 54.0% to 89.1%. Laser texturing processes were carried out in air atmosphere. When the laser irradiated Ti6Al4V along the laser scanning path, the materials in laser-irradiated regions were removed and the structures consisting of microgrooves were generated. The unprocessed regions remained and were protruding. The width of microgrooves was controlled by tuning the number of laser scanning lines with equidistant distance between adjacent lines, while the depth of microgrooves was controlled by tuning the laser scanning repetitions. After laser texturing process, the specimens were ultrasonically cleaned for 15 min using deionized water. The width and depth of the laser-fabricated microgrooves were measured through optical microscopy (Figure 2) and scanning electron microscopy (SEM, Figure 4).

### 2.3. Laser Joining Process

Before laser joining, the titanium alloy specimen was placed on top of CCF30/PEEK with the structured titanium alloy surface contacting with CCF30/PEEK. The lap width between titanium alloy and CCF30/PEEK is set as 9 mm (Figure 3). Two preset Ti6Al4V clamping plates perpendicular to the lap width direction were applied to provide pressure on the joining area (Figure 3b), and the pressure was controlled by turning the bolts. In order to ensure the close contact of two sheets, the fastening force of about 2.8 MPa was applied. Of note that the center of upper clamping plate was cut off so that the laser beam could be directly irradiated on the joined metal surface, as illustrated in Figure 3a. The laser joining process was carried out using a continuous laser scanning system with the beam wavelength of 1030 nm. Table 4 shows the main laser parameters for joining Ti6Al4V and CCF30/PEEK. When the beam irradiates the Ti6Al4V surface, laser energy is absorbed by metal surface and conducted through Ti6Al4V to heat the joining interface. The heat input near the lap interface was controlled by changing laser irradiation duration.

### 2.4. Property Characterizations

Surface morphology of the laser-fabricated microstructures on titanium alloy and the fractured surfaces of the joints was characterized by scanning electron microscopy (SEM, JSM-6610LV, JEOL, Tokyo, Japan). After laser joining process, cross-sectional microstructures near the joining interface were characterized using SEM. The strength of the produced joints between Ti6Al4V and CCF30/PEEK was tested by an electronic universal testing machine (Instron 5982, Instron, Boston, MA, USA). Two pads of the same thickness as the CCF30/PEEK and Ti6Al4V plates were tabled on both ends of the sample for alignment during the tests. The tests were carried out at a traveling speed of 1 mm/min. Five samples in each joining condition were prepared for shear strength testing to reduce the experimental error.

## 3. Results and Discussion

In the laser joining process, the top surface of Ti6Al4V specimen is continuously heated through laser irradiation induced thermal field, where the maximum temperature on the Ti6Al4V surface is approximately 302 °C, as indicated in Figure S1. The heat accumulated on the Ti6Al4V surface is conducted through the specimen to heat the composites near the interface between Ti6Al4V and CCF30/PEEK. When the PEEK resins near the interface are melted, the melted resins will be squeezed into the microgrooves along with the carbon fibers wrapped in the resins, due to the pre-exerted fastening force on the lapping area. As laser beam moves away from the irradiated surface, the melted resins will cool and solidify, leading to the formation of mechanical interlocks at the joining interface. Since the textures composed of the microgrooves with same geometric dimensions have similar structural characteristics, the laser joining process-produced CCF30/PEEK-Ti6Al4V joints displayed similar interfacial morphologies when different patterns (0° grating, 90° grating and 0°/90° grid) were pre-textured on Ti6Al4V the surface, as shown in Figure 4, indicated by the cross-sectional morphologies of the typical CCF30/PEEK-Ti6Al4V joints with 0° gratings pre-textured on the Ti6Al4V surface. When the width of microgrooves was 225 ± 15 μm, all the microgrooves were fully filled by the melted composites, leading to good mechanical interlocking structures at the joint interface (Figure 4a). As the width of the microgrooves increased to 265 ± 10 μm and above, porous defects appeared near the interface, and the pore size increased with the width of microgrooves (Figure 4b–d). Thus, the filled melted composites inside microgrooves exhibited porous morphology. It was clearly observed that the filled materials in the microgrooves included PEEK resins and carbon fibers, and partial carbon fibers were exposed due to the loss of the resins that wrapped the fibers (Figure 4c). Moreover, crevices and voids were also clearly observed between impregnating tapes and between carbon fibers, due to the outflow of the PEEK resins among carbon fibers (Figure 4b–d). This also indicates that the melted composites below these crevices and voids had not fully filled into the microgrooves during laser joining process, which was related to the blocking effect of carbon fibers on the flow behaviors of the melted PEEK resins. The formation of these defects resulted in lower adhesion between impregnating tapes and carbon fibers, as well as poor mechanical interlocking structures near the joining interface.

**Figure 5:**
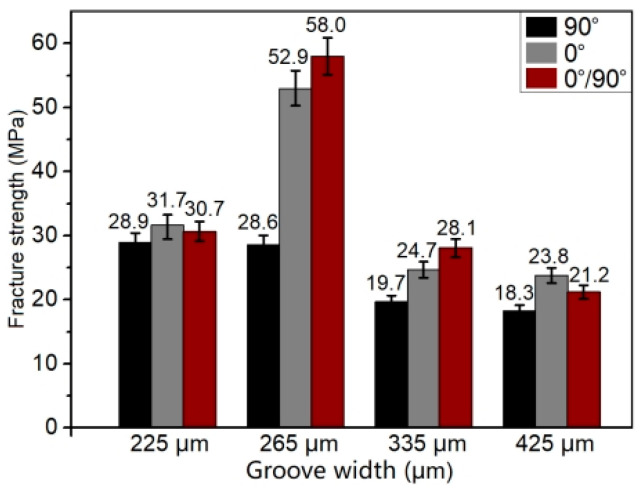
The fracture strength of the prepared CCF30/PEEK-Ti6Al4V joints with different textures on the Ti6Al4V surface.

Figure 5 shows the fracture strength of the produced CCF30/PEEK-Ti6Al4V joints with different patterns pre-textured on the Ti6Al4V surface, where the fracture strength of the joints was calculated by dividing the breaking force by the joining area. The results revealed that the microgroove orientation (0°/90°) of the grating textures on Ti6Al4V has an important influence on the fracture strength of the joints. When the microgroove orientation is consistent with the lapping direction (90°, Figure 2b), the fracture strength of joints is obviously higher than that of the joints with microgroove orientation perpendicular to the lapping direction (0°, Figure 2a), which may be related to the different stress distributions at the joining interface during tensile tests. Compared to the joints with grating textures on the Ti6Al4V surface, the joints with grid textures on the Ti6Al4V surface present slightly higher fracture strength, as shown in Figure 5. In the case of the microgroove width of 265 ± 10 μm, the joints achieved a maximum fracture strength of 58 MPa, which is higher than most previous reported results (10–40 MPa) of laser joined heterojunctions [19,20,21,29,30,31], as displayed in Figure 5. The higher joint strength was mainly attributable to the larger area of the mechanical interlocking structures and the strong adhesion at the joining interfaces. In contrast, when the width of microgrooves increased to above 265 ± 10 μm, numerous micropore defects (Figure 4c,d) led to poor mechanical interlocking structures and weak adhesion between Ti6Al4V and CCF30/PEEK near the joining interface, with the resultant joints having low fracture strength.

**Figure 6:**
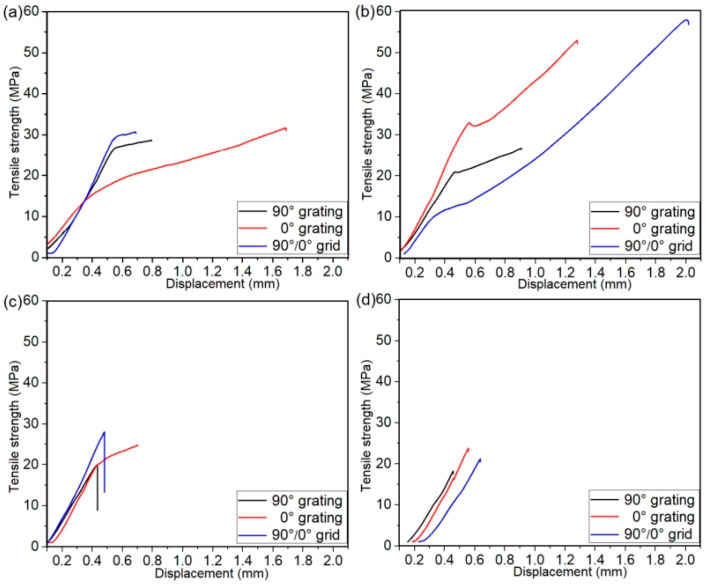
Tensile strength versus strain displacement curves of the CCF30/PEEK-Ti6Al4V joints with microgroove widths of 225 ± 15 μm (**a**), 265 ± 10 μm (**b**), 335 ± 10 μm (**c**), and 425 ± 15 μm (**d**), respectively.

Figure 6 shows the tensile stress versus strain displacement curves of the CCF30/PEEK-Ti6Al4V joints during tensile tests in the cases of various patterns pre-textured on Ti6Al4V surfaces. When the width of microgrooves was 225 ± 15 μm or 265 ± 10 μm, the tensile stress versus strain displacement curves during tensile tests were clearly divided into two distinct variation stages. Moreover, the growth rate of the tensile strength with displacement in the first stage was clearly higher than that in the second stage (Figure 6a,b). As the width of microgrooves increased to 335 ± 10 μm and 425 ± 15 μm, the tensile stress versus strain displacement curves displayed only the first stage. This indicated that the CCF30/PEEK joints fractured before the curves entered the second stage, which corresponded to the poor adhesion quality at the joining interface and the low fracture strength of the joints. Additionally, only when the width of microgrooves was 265 ± 10 μm, the joints underwent clear yield deformation stage during tensile tests, as shown in Figure 6b.

**Figure 7:**
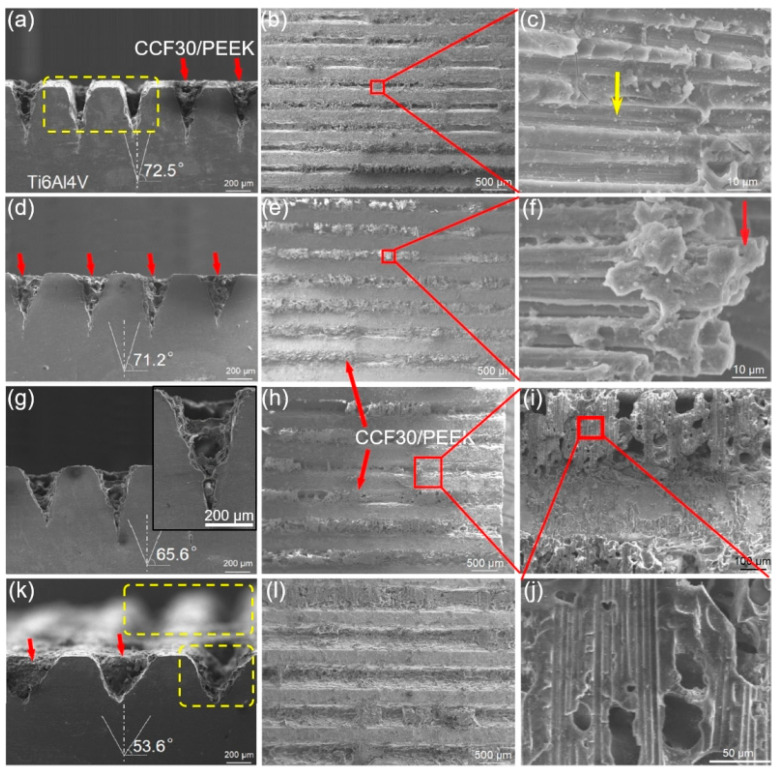
Fracture surface morphology of typical CCF30/PEEK-Ti6Al4V joints after tensile tests under the condition of microgroove orientation of 0°. The microgroove width was 225 ± 15 μm (**a**–**c**), 265 ± 10 μm (**d**–**f**), 335 ± 10 μm (**g**–**j**), and 425 ± 15 μm (**k**,**l**), respectively.

During tensile tests, all the Ti6Al4V-CCF30/PEEK joints fractured at the joining interface. Figure 7 shows the typical fractured morphologies on Ti6Al4V surfaces with 0° grating pre-textured on Ti6Al4V. In case of the microgroove width of 225 ± 15 μm (Figure 7a–c), most of the composites filled inside microgrooves were pulled out and the residual debris was mainly PEEK resins (Figure 7a,b), leaving traces of wrapping carbon fibers on the residual resin matrix, as shown in Figure 7c. Thus, the fracture modes of the joints in such a case included interface shear fracture and cohesive failure. In the case of the microgroove width of 265 ± 10 μm (Figure 7d–f), the failure of joints occurred mainly by interface shear fracture as presented in Figure 7d, and the breakage of carbon fibers was observed on the residual composites embedded inside the microgrooves (Figure 7f). When the microgroove width increased to 335 ± 10 μm and 425 ± 15 μm, the carbon fibers filled in the microgrooves were easily pulled out during tensile tests, due to the poor mechanical interlocking structures and the low adhesion at the joining interfaces between CCF30/PEEK and Ti6Al4V, leaving small amount of the resin debris on the Ti6Al4V surface (Figure 7g–l).

**Figure 8:**
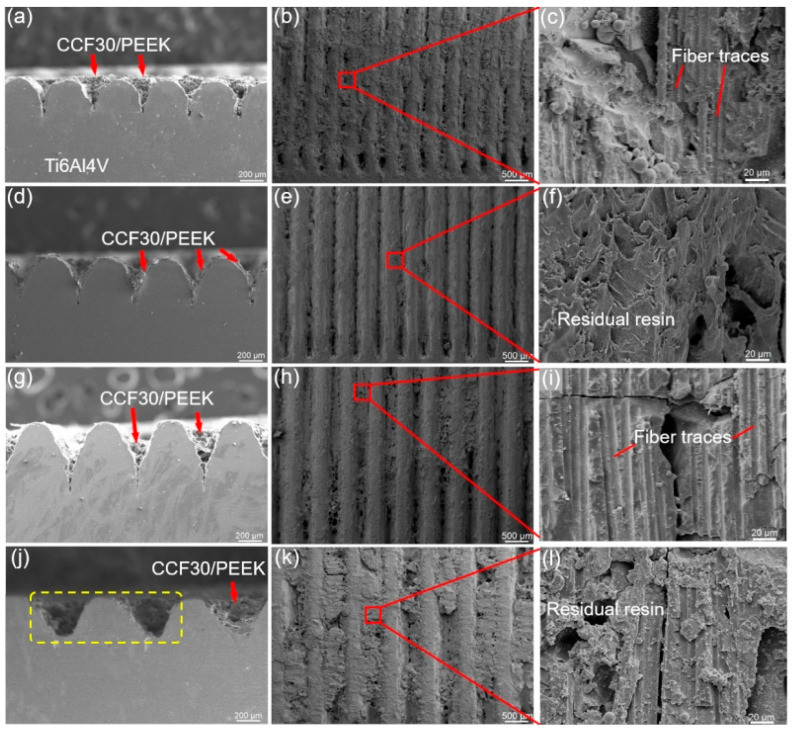
Fracture surface morphology of the typical CCF30/PEEK-Ti6Al4V joints after tensile tests under the condition of microgroove orientation of 90°. The microgroove width was 225 ± 15 μm (**a**–**c**), 265 ± 10 μm (**d**–**f**), 335 ± 10 μm (**g**–**i**), and 425 ± 15 μm (**j**–**l**), respectively.

Similarly, the typical fracture surfaces of CCF30/PEEK-Ti6Al4V joints with 90° gratings pre-textured on Ti6Al4V surface are displayed in Figure 8. From the morphologies of the fractured Ti6Al4V surfaces, it was observed that all the joints fractured mainly by cohesive failure, that is, the carbon fibers were detached from PEEK resins. In the case of the microgroove width of 225 ± 15 μm, fracture occurred near the joining interface with the majority of CCF30/PEEK composites remaining inside the microgrooves (Figure 8a–c) due to the formation of strong interlocking structures near the joining interface of joints. When the microgroove width was 265 ± 10 μm, the majority of the composites inside microgrooves were pushed out during tensile tests, leaving a small amount of resin residues at the bottom of the microgrooves (Figure 8d–f). In the cases of microgroove width of 335 ± 10 μm and 425 ± 15 μm, most of the porous-structured CCF30/PEEK composites were removed from microgrooves, leading to randomly distributed residual resin lumps on the fractured Ti6Al4V surface (Figure 8g–l).

**Figure 9:**
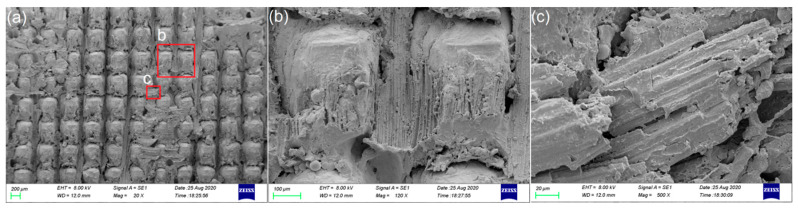
The morphology of fractured Ti6Al4V surfaces with 0°/90° grid patterns pre-textured on the Ti6Al4V surface. (**a**) SEM image showing the morphology of the fractured Ti6Al4V surface. (**b**) The residual PEEK resins in local area of the fractured Ti6Al4V surface. (**c**) The carbon fibers observed on the local fracture surface.

The fracture modes of the joints with grid structure on Ti6Al4V surface were similar to those with grating structures on Ti6Al4V, as observed in typical fracture morphology presented in Figure 9. After tensile tests, a large amount of resin matrix remained inside microgrooves, and numerous traces of the carbon fibers being pulled out were observed on the surface of residual resin (Figure 9b). Additionally, the fractured carbon fibers were locally observed on the fracture surface (Figure 9c).

## 4. Conclusions

In summary, the process of laser direct joining of titanium alloy and continuous carbon fiber-reinforced PEEK composites was demonstrated through constructing mechanical interlocking structures at the joining interface. Benefiting from the formation of interlocking structures and strong adhesion between CCF30/PEEK and Ti6Al4V, the maximum joint strength of over 58 MPa was achieved when pre-texturing grating or grid patterns on the Ti6Al4V surface with the optimized dimensions. However, various micro-defects, such as crevices and voids generated near the joining interfaces when microstructures with larger dimensions were pre-textured on the Ti6Al4V surface, which significantly reduced the fracture strength of the CCF30/PEEK-Ti6Al4V joints. During tensile tests, the failure of joints mainly occurred through interface shear fracture and cohesive failure, with the specific manifestations of the pulling out of carbon fibers from resin matrix and the breakage of carbon fibers. The presented results have explored the feasibility for joining continuous carbon fiber-reinforced composites with metals through laser joining technology and laid the foundation for achieving strong joining strength at the interfaces between dissimilar materials. Due to the low viscosity and poor flowability of PEEK resins in the molten state, as well as the hindering effect of the continuous carbon fibers on the melted resins, various micro-defects formed near the joining interface and it was difficult to construct stronger interlocking structures between CCF/PEEK and the metal via conventional laser joining process. Further exploration of the construction of high-performance interlocking structures between the metal and continuous fiber reinforced plastics is necessary to achieve heterojunctions with higher mechanical property.

## Figures and Tables

**Table 1:** Normal chemical compositions of titanium alloy Ti6Al4V.

Composition	C	Al	V	Ti
wt.%	≤0.1	5.88	3.46	Balance

**Table 2:** Main parameters of nanosecond laser micro-machining process.

Parameter	Value
Average output power, W	70
Pulse duration, ns	50
Pulse frequency, kHz	55
Travelling speed, mm/s	300
Wavelength, nm	1030

**Table 3:** Structural characteristics of laser machined patterns.

Characteristics	0° Grating Pattern				90° Grating Pattern				Grid Pattern			
	#1	#2	#3	#4	#1	#2	#3	#4	#1	#2	#3	#4
Width, μm	225 ± 15	265 ± 10	335 ± 10	425 ± 15	225 ± 15	265 ± 10	335 ± 10	425 ± 15	225 ± 15	265 ± 10	335 ± 10	425 ± 15
Distance, μm	400	450	500	700	400	450	500	700	400	450	500	700
Structure density, %	54.0	58.3	67.0	59.5	56.3	58.9	67.0	60.7	79.9	82.6	89.1	83.6

**Table 4:** Main parameters for laser joining of Ti6Al4V and CCF30/PEEK.

Parameter	Value
Average output power, W	300
Beam diameter, μm	35
Travelling speed, mm/s	2000
Wavelength, nm	1030
Laser type	Continuous fiber laser

## Data Availability

Data are contained within the article.

## Supplementary Materials

The following supporting information can be downloaded at: https://www.mdpi.com/article/10.3390/polym14214676/s1, Figure S1: Temperature distributions on laser irradiated titanium alloy surface during laser joining process.
