# Supplementary material for: Laser Joining of Continuous Carbon Fiber-Reinforced PEEK and Titanium Alloy with High Strength
Source: Polymers (Basel). 2022 Nov 2;14(21):4676. doi: 10.3390/polym14214676 (PMC9658059; doi:10.3390/polym14214676)
Supplement: Supplementary file 1 [file polymers-14-04676-s001.zip › polymers-1990047-supplementary.pdf]

polymers

MDPI

Supporting information

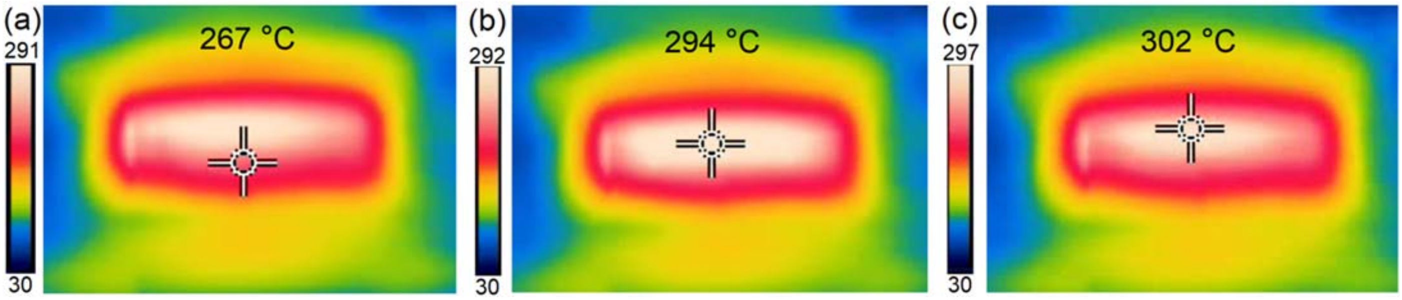

Figure S1. The temperature distributions of laser irradiated titanium alloy surface during laser joining process.
